# Does Eye Gaze Uniquely Trigger Spatial Orienting to Socially Relevant Information? A Behavioral and ERP Study

**DOI:** 10.3390/brainsci12091133

**Published:** 2022-08-25

**Authors:** Yichen Yuan, Jinqun Liu, Zehua Wu, Guomei Zhou, Werner Sommer, Zhenzhu Yue

**Affiliations:** 1 Guangdong Provincial Key Laboratory of Social Cognitive Neuroscience and Mental Health, Department of Psychology, Sun Yat-sen University, Guangzhou 510006, China; 2 Institut für Psychologie, Humboldt-Universität zu Berlin, 10099 Berlin, Germany; 3 Department of Psychology, Zhejiang Normal University, Jinhua 321004, China

**Keywords:** eye gaze, arrow, gaze cueing effect, attention orienting, threat-relevant targets

## Abstract

Using behavioral and event-related potential (ERP) measures, the present study examined whether eye gaze triggers a unique form of attentional orienting toward threat-relevant targets. A threatening or neutral target was presented after a non-predictive gaze or an arrow cue. In Experiment 1, reaction times indicated that eye gaze and arrow cues triggered different attention orienting towards threatening targets, which was confirmed by target-elicited P3b latency in Experiment 2. Specifically, for targets preceded by arrow and gaze cues, P3b peak latency was shorter for neutral targets than threatening targets. However, the latency differences were significantly smaller for gaze cues than for arrow cues. Moreover, target-elicited N2 amplitude indicated a significantly stronger cue validity effect of eye gaze than that of arrows. These findings suggest that eye gaze uniquely triggers spatial attention orienting to socially threatening information.

## 1. Introduction

During interpersonal communication, humans often use non-verbal signals, such as eye gaze, to convey intentions. Since gaze direction may indicate upcoming events at certain locations, one can infer others’ mental states from their gaze [1,2]. The gaze direction of others triggers gaze-following behavior of the observers and aligns spatial attention, that is, gaze direction facilitates joint attention [3,4,5]. Humans appear to be sensitive to eye gaze cues already in infancy, which may foster the development of joint attention [6,7,8].

People usually direct their attention to the locations gazed at by others, which facilitates the detection and processing of stimuli at that location. This is the basis of the gaze cueing effect, which has been widely investigated in the framework of Posner’s central cueing paradigm [9]. In this paradigm, eye gaze cues (i.e., eyes looking to the left or right) are presented at a (central) fixation marker; after an interval, targets are presented to the left or right side of the fixation marker. Usually, targets are detected more quickly when they appear at the cued (gazed-at) location than at the non-cued side. Facilitation by gaze cueing has been found for both schematic and real faces [10,11] and even if gaze direction does not validly predict target location [12]. These findings indicate that eye gaze is a strong attention cue, which automatically directs the observer’s attention towards the cued location.

Some researchers consider eye gaze as a unique social cue for attention and have demonstrated different neural mechanisms for attention orienting triggered by social gaze cues and non-social arrow cues [13,14,15,16,17,18,19], since gaze cues can facilitate joint attention. For example, Friesen et al. [15] used a central cueing paradigm to test the differences in attention orienting triggered by eye gaze and arrow cues. When a target was presented at the cued location with very low probability (cue predictiveness = 8%), eye gaze cues triggered attention orienting towards the cued location nevertheless; because this finding was not replicated by arrow cues, eye gaze-cued attention seems to be strongly reflex-like. In an event-related potential (ERP) study by Hietanen et al. [16], an early directing attention negativity was observed at 220–260 ms after the onset of arrow cues but not after gaze cues, suggesting that the attention orienting triggered by arrow cues was more voluntary and less reflexive than when triggered by gaze cues. Similarly, Langdon and Smith [17] found that reaction times (RTs) to targets preceded by invalid cues were significantly slower than to targets preceded by non-directional neutral cues in the gaze cue condition but did not differ in the arrow cue condition. These results suggest that gaze cues trigger stronger and longer reflexive attention orienting than arrow cues, possibly based on distinct mechanisms.

However, there is also evidence for similar attention mechanisms triggered by eye gaze and arrow cues [12,20,21,22,23]. For instance, Tipples [12] instructed participants to discriminate target letters in an arrow cueing paradigm and found that at cue-target intervals of 100 and 300 ms, non-predictive arrow cues also produced automatic orienting to the cued location. Brignani et al. [23] compared attention shifts induced by arrow and eye gaze cues with a purely endogenous cue, that is, a texture indicating target location. Their behavioral results showed a validity effect of texture cues only at a long stimulus-onset-asynchrony (SOA) of 700 ms; validity effects for arrow and gaze cues were present but indistinguishable at SOAs as short as 100 ms. Most importantly, functional microstate analysis showed similar topographical cortical maps for gaze and arrow cues, suggesting that similar cortical networks were involved (also see Tipper et al. [24]).

Recently, growing evidence has shown the uniqueness of eye gaze, compared to arrow cues. For instance, studies have found that gaze directs attention to specific locations, whereas arrows direct attention into a more general direction, spreading to the whole object in this direction [25,26]. Affective contexts are also shown to modulate the gaze cueing effect [27,28,29]. Dalmaso et al. [30] reviewed studies about affective contexts and concluded that social variables, such as the characteristics of participants or facially expressed emotions, could shape gaze-triggered attention orienting. Moreover, by adopting a spatial compatibility paradigm with gaze and arrows as targets, Marotta et al. [31] found standard spatial congruency effects for arrow cues; that is, faster responses to arrow targets when their location was compatible with their direction. However, a reversed congruency effect was observed for eye gaze cues. In their follow-up ERP study, Marotta et al. [32] found shared and dissociable congruency modulations for eye gaze and arrows. Specifically, they found similar effects for eye gaze and arrows at early processing stages (P1 and N1 components), whereas at later stages (N2 and P3 components) eye gaze and arrows showed opposite effects, underscoring the social characteristics of eye gaze.

Based on the previous findings, it seems eye gaze is unique to socially relevant information. For the gaze- and arrow-triggered orienting of attention, most previous studies focused only on the characteristics of the cue, e.g., affective contexts (such as emotional cues), experimental settings (such as SOA), and so on (for reviews see Dalmaso et al. [30] and Mckay et al. [5]). However, attention oriented by gaze and arrow cues always ends up with target processing and it is plausible that target characteristics might interact with the attention oriented by eye gaze or arrow cues [29,33,34]. For instance, by using (social) voice or (non-social) pure tones as targets, Zhao et al. [34] found that gaze cues induced stronger attention orienting to voice versus tone targets, compared with arrow cues. Their results indicate different attention orienting effects on targets when triggered by gaze or arrow cues, especially when cues and targets have congruent social meanings. However, voice targets, as used by Zhao et al. [34], can be affected by the individual experience, such as target interest. Among different social meanings, threatening information may be more salient and stable, because it may be more biologically grounded [35,36]. A study has also shown that threatening priming could enhance the gaze cueing effect but not the arrow cueing effect [37]. Thus, in the present study, threatening and neutral objects were used as targets. Moreover, previous studies examining the different attention orienting triggered by gaze and arrow cues adopted trial-by-trial or blocked designs, which might have different effects on the gaze and arrow cueing effect. Therefore, in the present study, we adopted randomized and blocked designs in Experiment 1 and 2, respectively, to explore the influence of experimental design on gaze- and arrow-triggered attention orienting.

The present study investigated whether eye gaze and arrow cues can trigger different forms of attention orienting. Moreover, we asked whether there is a unique form of attention orienting toward threat-relevant targets. In order to elucidate the neural correlates of spatial cueing, we also recorded ERPs in Experiment 2. In two experiments, size-standardized images of threatening or neutral objects were used as targets, to be judged in terms of their natural size relative to a fixed standard volume (a shoe box). Compared to the simple location detection tasks that are widely used in previous studies, this task requires the activation of conceptual, semantic properties of the object stored in long-term memory [38]. This task also requires the processing of target characteristics (threatening information), and has been used in studies of object recognition [39,40] and eye gaze cueing effects [29]. To further improve the ecological validity of the present research, dynamic eye gaze was adopted for the eye cues [27,41], that is, an initial direct eye gaze of the cue face was followed by a gaze shift to the left or right. A similar procedure was applied for the arrow cues.

For the behavioral performance, we expected a cueing effect for both gaze and arrow cues, that is, attention orienting to cued targets should be faster than to uncued targets. In addition, if the attention orienting process is modulated by the target characteristics, an interaction between cue type and target type should be observed. That is, compared to the arrow cues, the gaze cue should induce a stronger attention orienting towards threatening targets than towards neutral targets.

With respect to ERPs, we were interested in the target-elicited N1, N2, and P3b components. Specifically, N1 is the first negative deflection, peaking around 100 ms, which is widely used for indexing early attention allocation in object processing [42,43,44]. N2 is the second negativity, which is often observed at anterior and frontocentral locations; it is related to the discrimination of targets [45,46]. P3b is a long-lasting positivity, approximately peaking between 300 to 1000 ms after target onset. Previous research suggests that the magnitude of P3b amplitudes coincides with the threat level induced by images [47]. Moreover, the peak latency of P3b is related to task difficulty [48]. Thus, in the present study, we expected larger amplitudes after arrow cues than after gaze cues for the target-elicited N1 [16]. Since previous studies have found that eye gaze cues trigger stronger reflexive attention orienting relative to arrow cues [18], we expected larger amplitudes for the target-elicited N2 in validly than in invalidly cued trials [45,46]; this cue validity effect should be stronger when targets are preceded by eye gaze rather than arrow cues. For the target-elicited P3b, considering that responses to threatening targets are usually faster than to neutral targets [36,47,49], we expected earlier P3b peak latencies for threatening targets compared to neutral targets. Furthermore, significant modulation of attentional orienting on target processing should be expected at this stage. Compared to the arrow cues, the gaze cues should induce an earlier peak latency of P3b towards threatening targets than towards neutral targets.

## 2. Experiment 1

### 2.1. Methods

#### 2.1.1. Participants

A sample size calculation conducted using G*Power [50] revealed that to at least detect a significant interaction with effect size of $\eta_{p}^{2}$ = 0.275 (α = 0.05, 1 − β = 0.80), a sample of 23 participants was required. The expected effect size was taken from Zhao et al. [34], in which the independent variables and experimental design were similar to ours. In the present study, twenty-four healthy college students (15 females; mean age = 20.63 years, *SD* = 1.95, range = 19 to 25 years) from Sun Yat-sen University, Guangzhou, participated in the experiment. They reported no history of neurological or psychiatric disorders and had normal or corrected-to-normal vision. Participants signed informed consent and were paid 20 RMB. The study was approved by the Ethics Committee of the Department of Psychology, Sun Yat-sen University.

#### 2.1.2. Stimuli

**Figure 1:**
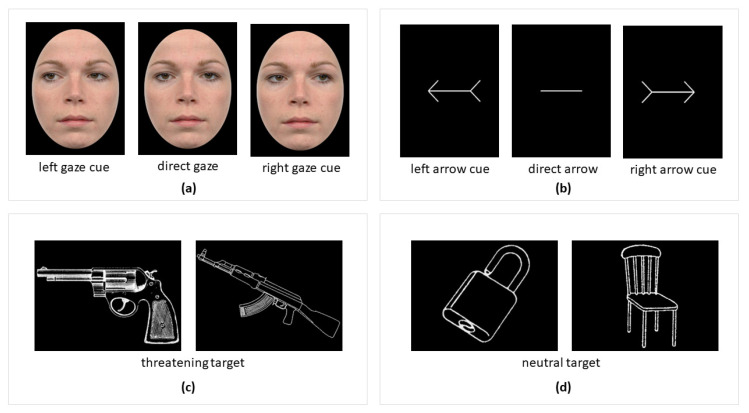
Illustration of the stimuli in the experiment. We used gaze (**a**) and arrow (**b**) stimuli as cues, and threatening objects (**c**) and neutral objects (**d**) as targets.

Portraits of 10 individuals with neutral expressions were selected from the Radboud Face Database [51]. Each picture was cropped by an elliptical frame of 6° × 8° to exclude hair, clothing, and background information, using Adobe Photoshop CC (San Jose, CA, USA). There were three versions of each portrait: looking straight ahead, to the left, and to the right (see Figure 1a). For the gaze-cue condition, the change in gaze direction was used as a spatial cue. For the arrow-cue condition, a horizontal arrow with the same length as the distance between the two pupils was used, with the arrowhead at one side and the tail at the other (see Figure 1b).

Pictures of 64 objects were obtained from Weldon and Roediger [52] and Internet sources. Another group of 27 raters classified each object as either neutral or threatening (binary), rated the level of arousal for each object on a 5-point Likert scale (1 = no arousal, 5 = highest arousal level), and judged whether or not in reality the depicted object would fit inside a box placed visibly next to the screen. Sixteen threatening and 16 non-threatening objects were selected: threatening objects such as a gun, a sword, a knife, etc., and neutral objects including a chair, a table, a book, etc. (see Figure 1c,d). The list of all objects used is provided in Appendix A. The arousal level was significantly higher for threatening than for neutral objects (*M* = 3.97 vs. 2.27, *SD* = 0.38 vs. 0.17), *t* (30) = 16.31, *p* < 0.001.

#### 2.1.3. Design and Procedure

The experiment was controlled by E-prime 2.0 software (http://www.pstnet.com/, Sharpsburg, PA, USA, accessed on 1 October 2015). Participants sat in front of a 23-inch monitor (1920 × 1080; 60 Hz) at a distance of 60 cm. A box sized 15 × 25 × 35 cm was placed next to the screen in sight of the participant. Prior to the experiment proper, participants judged whether each object would fit inside the box and received feedback.

**Figure 2:**
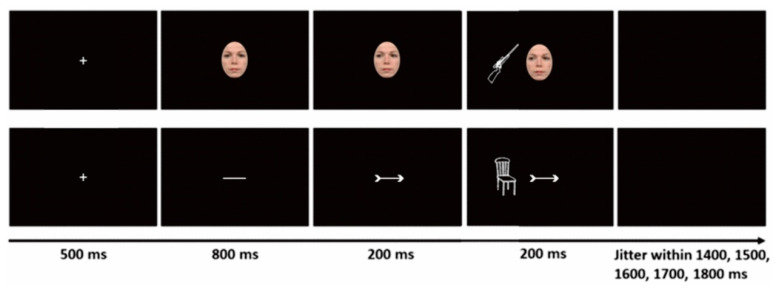
Schematic presentation of the experiment procedure ((**Top**): gaze-cue trial; (**bottom**): arrow-cue trial).

The trial scheme is shown in Figure 2. Each trial started with a fixation cross (1° of visual angle) presented at the center of the screen for 500 ms. The fixation cross was replaced either by a face (6° × 8° of visual angle) with direct eye gaze or by a horizontal line, presented for 800 ms. Then, the face shifted its gaze direction from direct to left or right averted and a left- or right-pointing arrowhead (plus corresponding tail) was added to the horizontal line. To avoid the possible overlap of cue-offset and target-elicited ERPs, the cue stimuli remained on the screen until the end of target presentation. Targets were presented 200 ms after cue onset, with equal probability, at the left or right side of the cue at an eccentricity of 6° (screen center to object center) and covering visual angles of 8° × 6° (width × height). After another 200 ms, both cue and target disappeared. Participants were required to judge whether or not in reality the depicted target object would fit inside the box and were advised to fixate on the center of the screen and avoid any horizontal or vertical eye movements during the trial. The next trial started 1400, 1500, 1600, 1700, or 1800 ms after target offset, with intervals selected at random and equiprobably. The experiment consisted of 10 blocks with 64 trials each. The eight conditions were equally assigned to each block, that is, both eye gaze and arrow cues were used in each block (within-block design). In addition, there were 16 practice trials. Thus, the present experiment employed a within-participant design with factors Cue type, Cue validity, and Target type.

#### 2.1.4. Data Analysis

Reaction times (RTs) and accuracy rates (ACC) were calculated separately for each experimental condition. Only RTs of correct responses between 100 and 1500 ms were included in the analysis, which led to the removal of 943 trials. In addition, RTs exceeding ± 2.5 *SD* of each participant’s mean RT were removed as outliers (1409 trials). Thus, 13,008 trials (85% of all trials) remained in the final data set, which yielded nearly 70 trials for each condition and each participant. ACC was the percentage of correct responses in the size classification task. Analyses of variance (ANOVAs) were calculated separately for RTs and ACC, including within-participant factors Cue type (arrow vs. eye gaze), Cue validity (valid vs. invalid), and Target type (threatening vs. neutral). All post-hoc comparisons were Bonferroni-corrected.

### 2.2. Results

**Figure 3:**
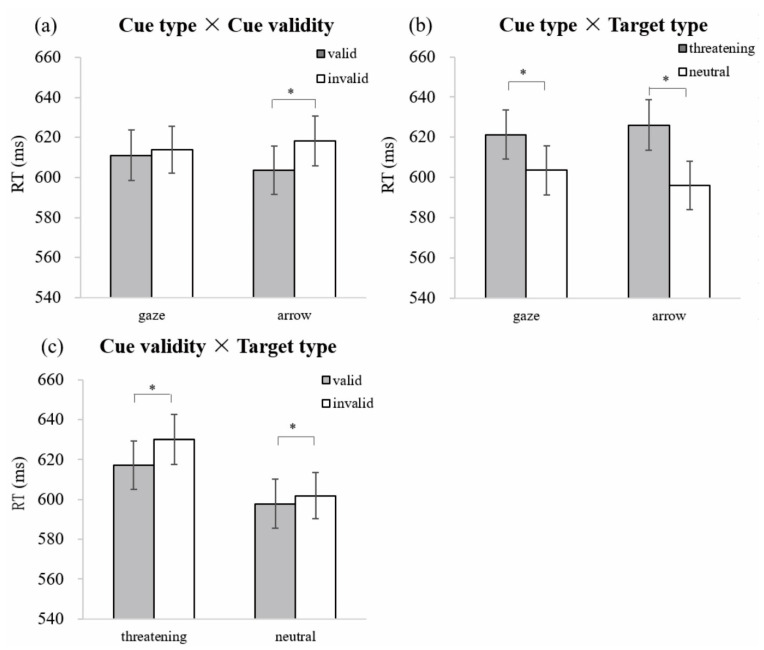
Mean RTs and standard errors for the interactions of (**a**) Cue type and Cue validity; (**b**) Cue type and Target type; (**c**) Cue validity and Target type. * *p* < 0.05. Error bar represents standard error.

Table 1 shows the behavioral performance in Experiment 1. To test whether gaze cue triggered a unique attention orienting to socially threatening targets, we ran ANOVAs with factors Cue type (arrow vs. eye gaze), Cue validity (valid vs. invalid), and Target type (threatening vs. neutral). For RTs, ANOVA revealed a significant main effect of Cue validity [*F*(1,23) = 20.53, *p* < 0.001, $\eta_{p}^{2}$ = 0.47], indicating that RTs to validly cued targets were shorter than to invalidly cued targets (*M* = 607 vs. 616 ms, *SE* = 12.09 vs. 11.88). The main effect of Target type was also significant [*F*(1,23) = 33.80, *p* < 0.001, $\eta_{p}^{2}$ = 0.60], suggesting faster responses to neutral than to threatening targets (*M* = 600 vs. 624 ms, *SE* = 11.95 vs. 12.29). Although there was no three-way interaction of Cue type, Cue validity and Target type [*F*(1,23) = 0.27, *p* = 0.611], the two-way interaction between Cue type and Cue validity was significant [*F*(1,23) = 7.03, *p* = 0.014, $\eta_{p}^{2}$ = 0.23]. Further analyses showed a cue validity effect in the arrow condition (*M* = 604 vs. 618 ms, *SE* = 12.04 vs. 12.37) [*t*(23) = 5.22, *p* < 0.001], but not in the eye gaze condition (*M* = 611 vs. 614 ms, *SE* = 12.50 vs. 11.75) [*t*(23) = 0.86, *p* = 0.396] (see Figure 3a).

In addition, the interaction between Cue type and Target type was significant [*F*(1,23) = 9.66, *p* < 0.005, $\eta_{p}^{2}$ = 0.30]. Follow-up analyses showed that RTs to threatening targets were significantly longer than to neutral targets; this was the case after both eye gaze cues (*M* = 626 vs. 596 ms, *SE* = 12.54 vs. 12.13) [*t*(23) = 6.59, *p* < 0.001] as well as arrow cues (*M* = 621 vs. 604 ms, *SE* = 12.38 vs. 12.11) [*t*(23) = 3.91, *p* = 0.001]. However, the difference in RTs between the threatening and neutral targets was significantly smaller in the eye gaze than in the arrow condition (*M*_diff_ = 18 vs. 30 ms, *SE* = 4.55 vs. 4.54) [*t*(23) = 3.11, *p* = 0.005], suggesting different attention orienting processes triggered by eye gaze and arrow cues (see Figure 3b).

Moreover, the interaction between Cue validity and Target type was significant [*F*(1,23) = 5.47, *p* = 0.028, $\eta_{p}^{2}$ = 0.19]. Further analyses revealed a cue validity effect (valid vs. invalid) for both threatening targets (*M* = 617 vs. 630 ms, *SE* = 12.24 vs. 12.56) [*t*(23) = 4.03, *p* = 0.001] and neutral targets (*M* = 598 vs. 602 ms, *SE* = 12.32 vs. 11.66) [*t*(23) = 2.12, *p* = 0.045]; however, the cue validity effect was significantly larger for threatening than for neutral targets (*M*_diff_ = 13 vs. 4 ms, *SE* = 3.23 vs. 1.97) [*t*(23) = 2.34, *p* = 0.028] (see Figure 3c).

For ACC, there was a significant main effect of Target type [*F*(1,23) = 63.28, *p* < 0.001, $\eta_{p}^{2}$ = 0.73], indicating that ACC for neutral targets was significantly higher than for threatening targets (*M* = 96.7 vs. 92.0%, *SEs* = 0.01). Neither the three-way interaction [*F*(1,23) = 0.01, *p* = 0.922] nor any of the two-way interactions [Cue type × Cue validity: *F*(1,23) = 0.31, *p* = 0.582; Cue type × Target type: *F*(1,23) = 0.90, *p* = 0.353; Cue validity × Target type: *F*(1,23) = 0.31, *p* = 0.583] were significant.

### 2.3. Discussion

In Experiment 1, we investigated whether gaze cues triggered a unique attention orienting compared to arrow cues, especially when they were directed towards socially threatening targets. Although responses to threatening targets were significantly slower than to neutral targets after both eye gaze and arrow cues, this effect was significantly smaller in the eye gaze cue condition than in the arrow cue condition. Moreover, although a significant overall cue validity effect was observed for the size classification of both threatening and neutral targets, this effect was stronger for threatening than neutral targets.

However, it should be noted that we did not find a significant gaze-specific cueing effect (interaction of Cue type × validity or Cue type × target type) in Experiment 1, which may be due to the randomized design mixing the effect of gaze cue and arrow cue and precluding cue-specific preparatory or attentional strategies. Thus, in an attempt to dissociate the effect of gaze and arrow cues, we adopted a block design in Experiment 2, allowing for the adoption of more specific preparation and expectation processes with respect to the targets, based on the type of cue. Moreover, to determine the neural correlates of gaze and arrow cues for threatening as compared to neutral targets, we recorded ERPs in Experiment 2. In contrast to Experiment 1, we expected more pronounced processing of threat-related targets on the behavioral level or at least on the neural level.

## 3. Experiment 2

### 3.1. Methods

#### 3.1.1. Participants

A sample size calculation conducted using G*Power [50] revealed that to at least detect a significant interaction with effect size of $\eta_{p}^{2}$ = 0.23 (taken from Zhao et al. [34], α = 0.05, 1 − β = 0.80), a sample of 29 participants was required. Considering that highly noisy ERP data had to be excluded, in the present study, forty healthy college students from Sun Yat-sen University, Guangzhou, were recruited. Data of ten individuals were excluded because of excessive (>25%) EEG artifacts. Hence, data of 30 participants (18 females; mean age = 19.4 years, *SD* = 1.38, range = 18 to 25 years) entered the final analysis. They reported no history of neurological or psychiatric disorders and had normal or corrected-to-normal vision. Participants signed informed consent and were paid 60 RMB. The study was approved by the Ethics Committee of the Department of Psychology, Sun Yat-sen University.

#### 3.1.2. Stimuli

The stimuli used in this experiment were the same as in Experiment 1.

#### 3.1.3. Design and Procedure

In Experiment 2, a within-participants design with factors Cue type (eye gaze vs. arrow), Cue validity (valid vs. invalid), and Target type (threatening vs. neutral) was adopted. Eye gaze cue and arrow cue conditions were implemented in separate blocks (between-block design), that is, only one type of cue was used in each block. There were six eye gaze cue blocks and six arrow cue blocks with 80 trials in each block. The trial sequence of Experiment 2 was the same as Experiment 1 (see Figure 2).

#### 3.1.4. Electroencephalogram (EEG) Recording and Preprocessing

The EEG was recorded from 64 Ag-AgCl electrodes mounted in an elastic cap (Easy Cap, Woerthsee-Etterschlag, Germany) with a NeuroScan SynAmps2 Amplifier (Scan 4.5, Neurosoft Labs, Inc., Sterling, VA, USA). A left earlobe electrode was used as an online reference. The ground electrode was located on the forehead. Vertical eye movements were monitored with an electrode below the right eye. Horizontal eye movements were recorded with two electrodes placed at the outer canthi of each eye. Electrode impedance was kept below 5 kΩ for all electrodes. Online recordings were band pass filtered at 0.05–100 Hz (12 dB/oct, 40 dB/dec) and sampled at 500 Hz. During the experiment, participants were instructed to maintain fixation at the center of the monitor and not to make horizontal eye movements.

**Figure 4:**
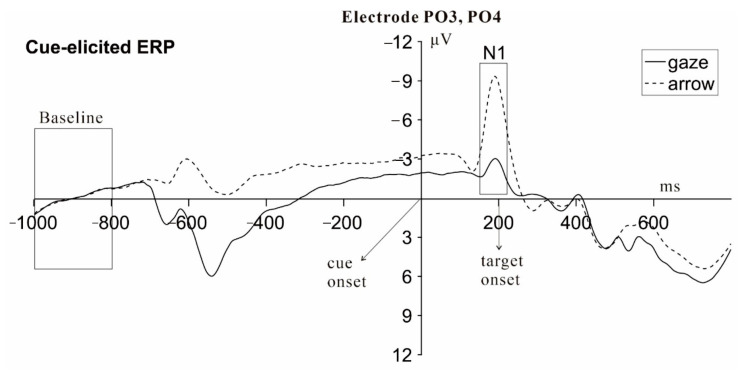
Grand-average cue-synchronized ERP waveforms for gaze and arrow cue conditions. Highlighted (right box) are the N1 components (averaged across electrodes PO3, PO4). Time zero on the *x*-axis corresponds to cue onset. The baseline was chosen at −1000 to −800 ms before the cue, corresponding to the 200 ms before the onsets of the horizontal line and face with direct gaze.

The offline analysis of EEG data was performed using Matlab R2016b (Natick, MA, USA, accessed on 1 May 2019) and eeglab14.1.2b (https://www.mathworks.com/, La Jolla, CA, USA, accessed on 15 March 2004). First, all scalp electrodes were re-referenced to the average of left and right earlobes. Then an infomax independent component analysis (ICA) algorithm [53] was applied for correcting eye movement artifacts. The SASICA plugin with ADJUST was used to identify the artifact component. Furthermore, considering the strongly different stimuli that will turn into cues (lines without arrow head vs. face with direct gaze), we chose a 200 ms baseline preceding these stimuli (−1000 to −800 ms prior to the cues and −1200 to −1000 ms prior to the targets; Figure 4) for both cue-elicited and target-elicited ERPs; EEG signal epochs ended 800 ms after the onset of the target stimuli. Finally, trials with voltages exceeding ±100 μV were excluded from ERP averages. The average artifact rejection rate was 7.3% of all trials (range 0 to 19.0%, remaining trials in each experimental condition: 84–120 trials). ERPs to eye gaze and arrow cues, and threatening and neutral targets were averaged separately for each participant.

#### 3.1.5. Data Analysis

Behavioral data analysis was conducted in the same way as in Experiment 1. For RTs, 26,282 trials (91% of all trials) remained in the final data set, which yielded nearly 110 trials for each condition and each participant.

For the ERPs elicited by cue stimuli, the mean amplitudes of N1 were measured in the time window of 150–220 ms after cue onset at electrodes PO3 and PO4, as suggested by previous studies [54,55]. Mean amplitudes were analyzed using ANOVAs with repeated measures on factors Cue type (eye gaze vs. arrow) and Hemisphere (left vs. right).

For the ERPs elicited by target stimuli, the mean amplitudes of N1 (110–220 ms) and N2 (280–380 ms) and the peak latency of P3b (450–700 ms) were analyzed. N1 was measured at electrodes F1 and F2, as suggested by previous studies [56,57], and N2 was measured at electrodes FC1 and FC2 [46]. Peak latency of P3b was computed for the centro-parietal electrodes CP1 and CP2 as suggested by Miller and Martin [47]. Target-elicited ERPs were analyzed using repeated measures ANOVAs with factors Cue type (eye gaze vs. arrow), Cue validity (valid vs. invalid), Target type (threatening vs. neutral), and Hemisphere (left vs. right). If necessary, the Greenhouse-Geisser method was adopted to correct degrees of freedom. In addition, Bonferroni corrections were used for post-hoc pair-wise comparisons and simple effects.

### 3.2. Results

#### 3.2.1. Behavioral Performance

To test whether gaze cue triggered a unique attention orienting to socially threatening targets, we ran an ANOVA with factors Cue type (arrow vs. eye gaze), Cue validity (valid vs. invalid), and Target type (threatening vs. neutral). For RTs, ANOVA revealed a significant main effect of Cue type [*F*(1,29) = 10.99, *p* = 0.002, $\eta_{p}^{2}$ = 0.28], indicating slower responses to target stimuli preceded by eye gaze cues as compared to arrow cues (*M* = 620 vs. 602 ms, *SE* = 11.61 vs. 9.89). The main effect of Cue validity was significant [*F*(1,29) = 6.32, *p* = 0.018, $\eta_{p}^{2}$ = 0.18], indicating faster responses to validly than to invalidly cued targets (*M* = 609 vs. 613 ms, *SE* = 10.38 vs. 10.52). In addition, there was a strong main effect of Target type [*F*(1,29) = 77.43, *p* < 0.001, $\eta_{p}^{2}$ = 0.73] since responses to threatening targets were slower than to neutral targets (*M* = 628 vs. 593 ms, *SE* = 10.99 vs. 10.22). Neither the three-way interaction [*F*(1,29) = 0.38, *p* = 0.540] nor any two-way interactions [Cue type × Cue validity: *F*(1,29) = 0.05, *p* = 0.821; Cue type × Target type: *F*(1,29) = 0.06, *p* = 0.816; Cue validity × Target type: *F*(1,29) = 0.08, *p* = 0.785] were significant.

For ACC, there was a significant main effect of Target type [*F*(1,29) = 41.20, *p* < 0.001, $\eta_{p}^{2}$ = 0.59], indicating that ACC for neutral targets was significantly higher than for threatening targets (*M* = 96.9 vs. 92.2%; *SE* = 0.004 vs. 0.01). We also found a significant interaction between Cue validity and Target type [*F*(1,29) = 5.10, *p* = 0.032, $\eta_{p}^{2}$ = 0.15]. Further analyses showed that for threatening targets, ACC was significantly higher in the valid than in the invalid cue condition (*M* = 92.5 vs. 91.8%, *SEs* = 0.01) [*t*(29) = 3.73, *p* = 0.032, one-tailed]. By contrast, for neutral targets, there was no significant difference in ACC between the valid and the invalid cue condition (*M* = 96.7 vs. 97.0%, *SEs* = 0.01) [*t*(29) = 1.50, *p* = 0.152]. The other two-way interactions [Cue type × Cue validity: *F*(1,29) = 0.002, *p* = 0.961; Cue type × Target type: *F*(1,29) = 0.64, *p* = 0.429] and the three-way interaction [*F*(1,29) = 1.19, *p* = 0.284] were not significant.

#### 3.2.2. Cue-Elicited ERPs (N1, 150–220 ms)

The grand-average ERP waveforms of N1 elicited by gaze and arrow cues at PO3 and PO4 electrodes are shown in Figure 4. To compare the attention orienting triggered by eye gaze and arrow cues, we ran an ANOVA of cue-elicited N1 amplitudes with factors Cue type (arrow vs. eye gaze) and Hemisphere (left vs. right). This ANOVA revealed a significant main effect of Cue type [*F*(1,29) = 40.39, *p* < 0.001, $\eta_{p}^{2}$ = 0.58] and Hemisphere [*F*(1,29) = 10.52, *p* = 0.003, $\eta_{p}^{2}$ = 0.27]. The interaction between Cue type and Hemisphere was significant [*F*(1,29) = 13.95, *p* = 0.001, $\eta_{p}^{2}$ = 0.32]. Further analyses showed that N1 amplitude was more negative to arrow than gaze cues at both PO3 and PO4 electrodes [PO3: *F*(1,29) = 40.33, *p* < 0.001; PO4: *F*(1,29) = 34.76, *p* < 0.001]. This Cue type effect was significantly larger at electrode PO3 than PO4 [*t*(29) = 3.73, *p* = 0.001].

#### 3.2.3. Target-Elicited ERPs

**Figure 5:**
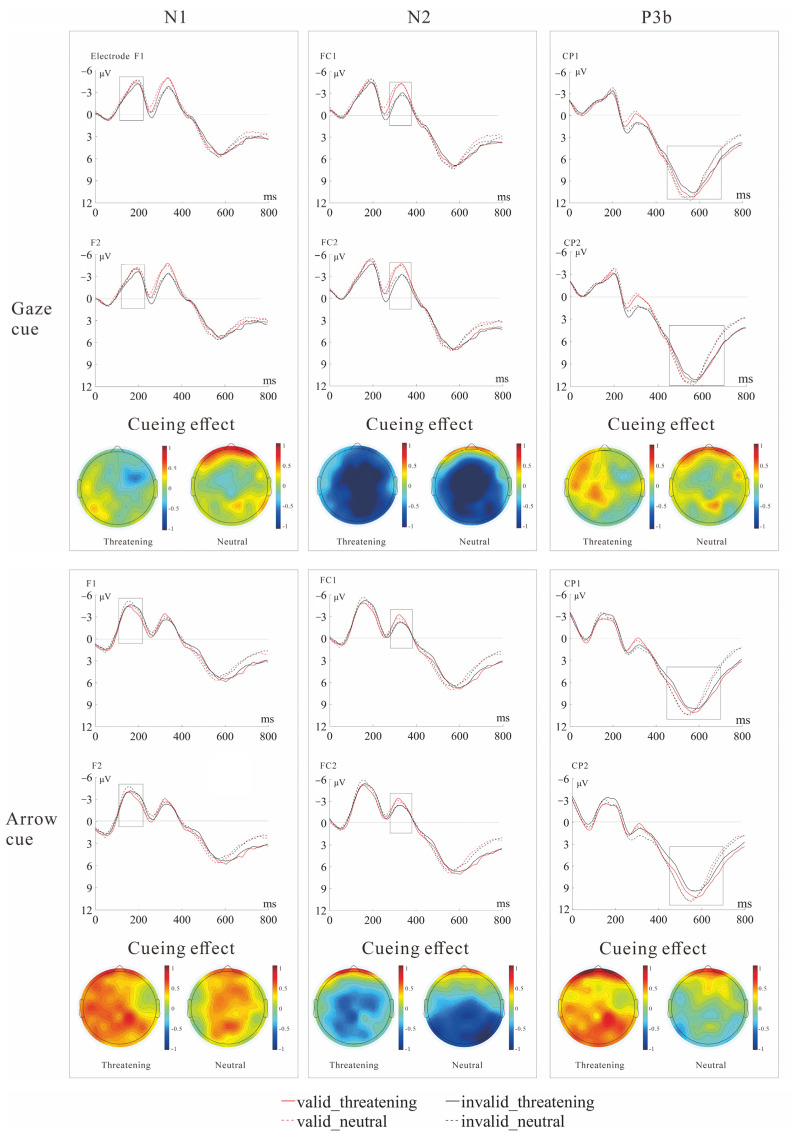
Grand-average target-synchronized ERP waveforms at selected electrodes for each experimental condition. Highlighted are the N1 (electrodes F1, F2, **left**), N2 (electrodes FC1, FC2, **middle**), and P3b (electrodes CP1, CP2, **right**) components. Time zero on the *x*-axis corresponds to target stimulus onset. The topographies beneath the waveforms show the gaze or arrow cue validity effect (valid–invalid) to threatening and neutral targets, respectively.

**Figure 6:**
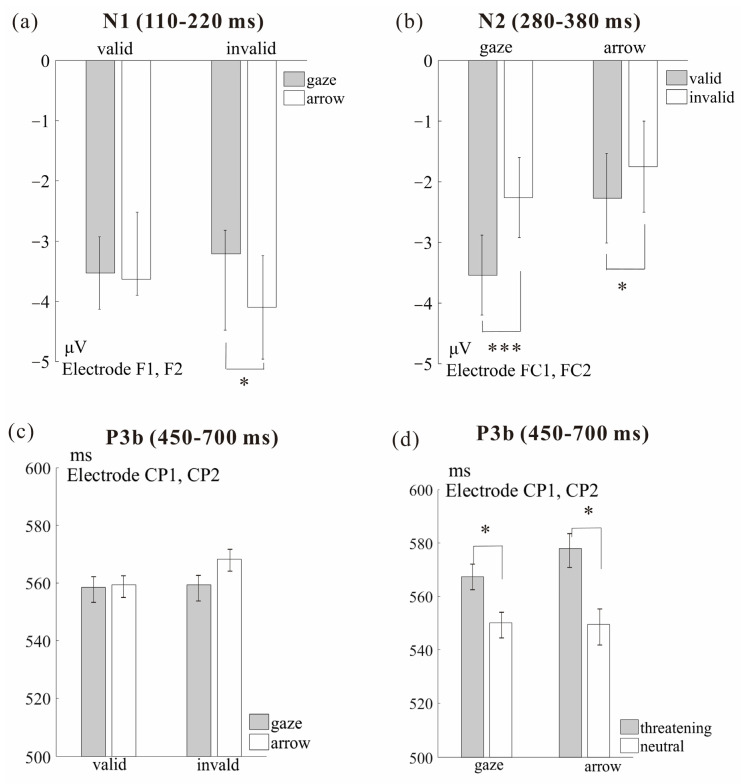
Average amplitudes and standard errors of the target-elicited (**a**) N1 (110–220 ms), (**b**) N2 (280–380 ms). (**c**,**d**) Mean peak latency and standard errors of the target-elicited P3b (450–700 ms) for the interactions of (**c**) Cue type and Cue validity and (**d**) Cue type and Target type. * *p* < 0.05. *** *p* < 0.001.

To investigate the underlying neural mechanism in gaze- and arrow- triggered attention orienting toward socially threatening targets, we ran ANOVAs separately for the interested components, namely, the mean amplitudes of the N1 and N2, and the peak latencies of P3b. Figure 5 shows the grand-average ERPs to targets for each experimental condition at electrodes that highlight the N1, N2, and P3b components. The mean amplitudes of the N1 and N2, the peak latencies of P3b, and their standard errors can be seen in Figure 6.

**N1 (110–220 ms)**

ANOVA of N1 mean amplitudes was conducted with factors Cue type (arrow vs. eye gaze), Cue validity (valid vs. invalid), Target type (threatening vs. neutral), and Hemisphere (left vs. right). This ANOVA showed a significant interaction between Cue type and Cue validity [*F*(1,29) = 5.02, *p* = 0.033, $\eta_{p}^{2}$ = 0.15] (see Figure 6a). Follow-up analyses showed that N1 amplitude to targets following invalid arrow cues was more negative than following invalid gaze cues (*M* = −3.60 vs. −2.72 μV, *SE* = 0.78 vs. 0.65) [*t*(29) = 2.08, *p* = 0.047], indicating that attention is easier to allocate during the re-orienting process after arrow cues than after gaze cues. However, for targets preceded by valid cues, no significant difference in N1 amplitude was observed between gaze and arrow cues. No other significant main effect or interaction was found.

**N2 (280–380 ms)**

For N2, ANOVA was conducted with factors Cue type (arrow vs. eye gaze), Cue validity (valid vs. invalid), Target type (threatening vs. neutral), and Hemisphere (left vs. right). The N2 amplitude was more negative to targets preceded by gaze cues than arrow cues (*M* = −2.89 vs. −2.01 μV, *SE* = 0.65 vs. 0.74) (see Figure 6b), as confirmed by a significant main effect of Cue type [*F*(1,29) = 7.92, *p* = 0.003, $\eta_{p}^{2}$ = 0.22]. The main effect of Cue validity was also significant [*F*(1,29) = 20.65, *p* < 0.001, $\eta_{p}^{2}$ = 0.42], reflecting more negative N2 amplitudes to targets preceded by valid than by invalid cues (*M* = −2.90 vs. −2.00 μV, *SE* = 0.68 vs. 0.69).

Furthermore, the interaction between Cue type and Cue validity was significant [*F*(1,29) = 8.74, *p* < 0.001, $\eta_{p}^{2}$ = 0.23]. Further analyses showed that N2 amplitude was more negative to targets preceded by valid than invalid cues; this was the case after both eye gaze cues (*M* = −3.54 vs. −2.26 μV, *SE* = 0.66 vs. 0.66) [*t*(29) = 5.39, *p* < 0.001] and arrow cues (*M* = −2.27 vs. −1.75 μV, *SE* = 0.74 vs. 0.75) [*t*(29) = 2.20, *p* = 0.036]. However, the cue validity effect was significantly stronger when targets were preceded by gaze cues than by arrow cues [*t*(29) = −2.96, *p* = 0.006].

Moreover, the interaction between Cue type, Cue validity, Target type and Hemisphere was significant [*F*(1,29) = 7.33, *p* = 0.011, $\eta_{p}^{2}$ = 0.20]. Further analyses showed a significant three-way interaction between Cue validity, Target type, and Hemisphere for gaze cues [*F*(1,29) = 6.19, *p* = 0.019, $\eta_{p}^{2}$ = 0.18], but not for arrow cues [*F*(1,29) = 0.95, *p* = 0.337], indicating that a stronger cue validity effect was observed for gaze cues than arrow cues. For gaze cues, follow-up analysis showed a significant Cue validity by Hemisphere interaction only for neutral targets [*F*(1,29) = 4.41, *p* = 0.045, $\eta_{p}^{2}$ = 0.13], indicating that a reliable cue validity effect was found at the left hemisphere.

**P3b (450–700 ms)**

ANOVA of P3b peak latency was conducted with factors Cue type (arrow vs. eye gaze), Cue validity (valid vs. invalid), Target type (threatening vs. neutral), and Hemisphere (left vs. right). This ANOVA showed a significant main effect of Target type [*F*(1,29) = 41.68, *p* < 0.001, $\eta_{p}^{2}$ = 0.59], revealing that P3b latency was shorter for neutral targets than threatening targets (*M* = 549.88 vs. 572.66 ms, *SE* = 6.05 vs. 5.97).

Furthermore, the interaction between Cue type and Cue validity was significant [*F*(1,29) = 5.66, *p* = 0.024, $\eta_{p}^{2}$ = 0.16] (see Figure 6c). Follow-up analyses showed that for invalidly cued targets, there was a trend for P3b latency to be shorter in gaze cue than arrow cue conditions [*M* = 558.48 vs. 568.25 ms, *SE* = 5.41 vs. 7.24, *t*(29) = 1.81, *p* = 0.081]. By contrast, for targets preceded by valid cues, no significant difference in P3b latency was observed between gaze and arrow cue conditions [*M* = 559.03 vs. 559.30 ms, *SE* = 6.00 vs. 6.82, *t*(29) = 0.07, *p* = 0.943].

The interaction between Cue type and Target type was also significant [*F*(1,29) = 6.30, *p* = 0.018, $\eta_{p}^{2}$ = 0.18] (see Figure 6d). Further analyses showed that for targets preceded by arrow cues and gaze cues, P3b latency was shorter for neutral targets than threatening targets [arrow cue: *M* = 549.55 vs. 578.00 ms, *SE* = 7.12 vs. 7.47, *t*(29) = 5.81, *p* < 0.001; gaze cue: *M* = 550.20 vs. 567.32 ms, *SE* = 5.53 vs. 5.47, *t*(29) = 5.14, *p* < 0.001]. However, the latency differences between neutral and threatening targets were significantly larger for arrow cues than for gaze cues [*M* = 28.45 vs. 17.12 ms, *SE* = 4.90 vs. 3.33, *t*(29) = 2.51, *p* = 0.018], indicating that gaze cues facilitate the processing of threatening targets relative to arrow cues. On the other hand, for threatening targets, there was a trend for P3b latency to be shorter for gaze cues than arrow cues [*M* = 567.32 vs. 578.25 ms, *SE* = 5.45 vs. 7.47, *t*(29) = 1.98, *p* = 0.057]. By contrast, for neutral targets, no significant difference in P3b latency was observed between gaze and arrow cues.

### 3.3. Discussion

In Experiment 2, accuracy showed a significant interaction between Cue validity and Target type. That is, ACC in the valid cue condition was significantly higher than in the invalid cue condition for threatening targets but not for neutral targets, indicating that valid cues facilitate size classification especially for threatening targets. In addition, slower reaction times and lower ACC were found for threatening targets than for neutral targets in both experiments, indicating that participants require more time to judge the real-life size for threatening than for neutral targets. The ERPs of Experiment 2 showed that arrow and gaze cues activated different neural mechanisms of attentional orienting at both early (N1) and late (N2 and P3b) stages of target processing. The amplitude of the target-elicited N1 was more negative following invalid arrow cues than following invalid gaze cues, although the N1 amplitude to targets was the same after both arrow and gaze cues in response to validly cued targets (ruling out low-level stimulus effects). These results suggest that the direction information in arrow cues could be harder to disengage than that in gaze cues. Moreover, target-elicited P3b showed that eye gaze cues diminished the difference in P3b latency between threatening and neutral targets, indicating that gaze cues facilitate the processing of threatening targets relative to arrow cues. Interestingly, this facilitation by eye gaze appeared to be general and independent of the validity of the cues. Thus, the facilitation may come from seeing eye gaze cues as compared to arrow cues.

## 4. General Discussion

The purpose of the present study was to investigate the effect of gaze-triggered, relative to arrow-triggered, attention orienting on the processing of threat-relevant targets. In Experiment 1, behavioral results showed that responses to threatening targets were significantly slower than neutral targets; this was the case after both eye gaze cues and arrow cues. However, the difference in RTs between the threatening and neutral targets was significantly smaller in the eye gaze cue than in the arrow cue condition. Similarly, in Experiment 2, a significant interaction between Cue type and Target type was observed for the peak latency of target-elicited P3b (450–700 ms). The P3b latency elicited by threatening targets was shorter after gaze cues than after arrow cues. These results indicate that compared to arrows, eye gaze triggers a unique spatial orientation towards socially threatening targets, based on the access to object knowledge [38]. Moreover, in line with Experiment 1, behavioral results in Experiment 2 showed slower reaction times and lower ACC for threatening than for neutral objects. Here, a significant interaction between Cue validity and Target type was observed for ACC instead of RTs. That is, ACC in the valid cue condition was significantly higher than in the invalid cue condition; this was the case for threatening targets but not for neutral targets, indicating that valid cues could facilitate processing especially for threatening targets.

Although RTs or ACC did not indicate a differential effect of Cue type on Cue validity or Target type, the ERP findings revealed such an effect. In the ERPs of Experiment 2, cue-elicited and target-elicited ERPs showed that compared to arrow cues, eye gaze cues trigger different attention orienting towards upcoming targets. The cue-elicited N1 was larger for arrow than for eye gaze cues, which is inconsistent with our expectation. This result might be due to possible interference between the target objects and the eyes because they both share social characteristics. This interfered attention allocation might render the gaze cues less effective than arrow cues. Similarly, for target-elicited ERPs, different ERP results for eye gaze cues and arrow cues were observed both at early and late stages, as manifested in the N1 (110–220 ms), N2 (280–380 ms), and P3b (450–700 ms) components.

Firstly, our results suggest that attention could be easier to reallocate towards new locations after arrow cues than after gaze cues. In the invalid cue condition, the amplitude of the target-elicited N1 (110–220 ms) was larger to target stimuli preceded by arrow cues as compared to gaze cues. By contrast, such a cue-related difference in N1 amplitude was not present in the valid cue condition. These results indicate that both arrow and gaze cues can direct attention to targets efficiently during an early stage [23]; however, it is easier to shift attention towards a new spatial location when it is oriented by an arrow cue as compared to a gaze cue, which is consistent with previous studies [16,58]. There is also evidence showing that arrow cues are related to voluntary orienting whereas eye gaze cues are more related to reflexive orienting [16,59].

Secondly, our results showed that eye gaze cues could facilitate the identification of targets, especially of validly cued targets, as compared to arrow cues. For the target-elicited N2 waveforms (280–380 ms), ERP results revealed a significant interaction between Cue type and Cue validity. Larger N2 amplitudes were observed for targets preceded by valid cues than by invalid cues, echoing the main effect of validity in RTs, which were shorter in the valid cue condition than in the invalid cue condition. Importantly, the validity effect on N2 amplitude was significantly stronger in the eye gaze cue condition than in the arrow cue condition, an interaction that was not present in performance. Previous studies have shown that the N2 component observed at anterior and frontocentral locations is related to the identification of targets [45,46]. Therefore, the present ERP results suggest that valid cues facilitate object identification and processing, and this effect is more pronounced for social eye gaze cues. Considering that our task requires the access to conceptual knowledge about targets, the eye gaze cue seems to facilitate target knowledge retrieval and semantic processing. These findings are consistent with previous findings that gaze triggers stronger attentional orienting than arrows and facilitates the identification and processing of upcoming stimuli [10,15,18]. From an ecological perspective, the attentional orienting of eye gaze conveys important information for survival; hence, the identification and processing of upcoming targets cued by eye gaze may be evolutionarily prepared.

Finally, our results suggest that eye gaze cues are especially effective for late processing stages and facilitate the classification of threatening targets at late stages. There was a trend that P3b (450–750 ms) latency was shorter for gaze cues than arrow cues; this was the case in the invalid cue condition but not in the valid cue condition. These results indicate that gaze cues always facilitate the processing of targets even if the gaze cues are invalid. Compared to our N1 results, indicating that arrow cues could be more efficient in shifting attention towards new spatial locations, the P3b latency results indicate that gaze cues are especially effective for late processing stages. Although it might be more difficult for gaze cues to shift attention, it was easier to process and classify target size once the attention re-orienting was accomplished by gaze cues as compared to arrow cues.

For the peak latency of target-elicited P3b (450–700 ms), a significant interaction between Cue type and Target type was observed. P3b latency was shorter for neutral than for threatening targets in both arrow and gaze cue conditions. However, the latency differences between neutral and threatening targets were significantly smaller for eye gaze cues than for arrow cues. Hence, P3b latency elicited by threatening targets is shorter after gaze cues. P3b responses are generally considered not only to reflect the threat level of images [47] but also the classification speed of stimuli, with shorter latencies for easier and faster classification [60,61,62]. Therefore, our results suggest that gaze cues facilitate the classification of threatening objects. Specifically, for both arrow and gaze cues, the negative threatening targets elicited longer P3b latency compared to neutral targets, which might be related to their slower and less accurate classification performance. However, the classification of threatening targets was facilitated by social gaze cues, suggesting that relative to arrow cues, gaze cues might facilitate the recognition of threatening targets, but not neutral targets.

In addition, our P3b results highlight the necessity for taking the characteristics of targets into account when comparing attention orienting effects induced by social eye gaze and non-social arrow cues. From an ecological perspective, the fast detection of potential danger is crucial for human survival. Some studies have also indicated that gaze cueing effects can be attributed to the evolutionary advantage of detecting predators or other threats in the environment [63]. Thus, it is plausible that target characteristics (especially threat-relevant targets) can affect attention orienting induced by gaze and arrow cues. However, as far as we know, few studies have focused on the influence of target characteristics on attention orienting. In our previous study [29], we used a gaze cueing paradigm and observed larger amplitudes of late positive potential (LPP) when cue and target were emotionally congruent, i.e., a threatening animal cued by gaze of a fearful face. This result suggests that target characteristics may influence the effect of attention orienting of eye gaze and that the contextual congruence between cue and target may enhance the cueing effect. Thus, the present results further support that eye gaze is a special socially relevant cue, and the classification of threatening targets is facilitated by attention orienting triggered by gaze cues.

It should be noted that in the present study, threatening objects elicited longer RTs and P3b latencies than neutral targets, not indicating any threat superiority effect. This is inconsistent with some previous studies [47,49], which might be partly due to the specific targets used in the present study, line drawings. These targets may be less ecological compared with the full-color pictures used in other studies [36]. Furthermore, attention orienting may be affected by the familiarity of targets. Thus, Baskin-Sommers et al. [64] found that familiar stimuli elicited larger late positive potentials in the ERP than novel stimuli. Threatening objects (e.g., guns, swords, etc.) are not as often seen or expected in daily life as neutral objects (e.g., chairs and cups, etc.). Hence, the longer RTs and P3b latencies for threatening targets could be attributed to their lower familiarity relative to neutral objects. In addition, the emotional or arousal value of targets may also matter. For example, Zsido et al. [65] found modern threatening emotional stimuli (guns) were detected faster than evolutionarily relevant stimuli (snakes); however, this modern threat superiority effect was only found in stimuli with high but not with moderate arousal levels. Although in the present study the arousal level felt in response to threatening objects was significantly higher than for neutral objects, the arousal level for neutral objects was relatively high. Future studies might investigate whether non-social threats (snakes, spiders, scorpions, and so on) work differently. In addition, the characteristics of targets need to be further explored, such as the emotional or semantic information. Moreover, one may use various targets including social information to study the attention orienting triggered by eye gaze in special populations, e.g., individuals with autism spectrum disorders.

In the present study, a blocked design was adopted in Experiment 2, while all conditions had been randomized in Experiment 1. By using the same set of stimuli and trial sequences, our results might shed light on whether the experimental design would affect the strength of attention orienting triggered by gaze and arrow cues. From the current results, it seems that the blocked design is a better way to compare the attention orienting triggered by gaze and arrow cues. Previous studies also found that compared to the intermixed design, the blocked design might improve the effect size [25]. Considering the reduced gaze cueing effect with more demanding tasks [66,67,68], one possible explanation is that the trial-by-trial design potentially enhances the attentional demands of the task, because participants have to shift their attention focus between different perceptual features, namely the gaze and arrows [69].

As mentioned about the task demands, one might argue that it may reduce the gaze cueing effect [66,67,68]. In the present study, although a size classification task rather than a simple location detection task was adopted, we still found that gaze cues could promote the processing of upcoming targets, especially for threatening targets. This object size judgment task was also adopted in the previous eye gaze study, in which a significant gaze cueing effect was reported [29]. Moreover, our results revealed differential attention orienting for eye gaze and arrow cues, consistent with previous studies [10,17,18,19].

In summary, the present study investigated the different orienting mechanisms of eye gaze and arrows as cues, especially the effect of gaze-triggered orienting on the processing of threatening targets. Our results support that gaze cues uniquely trigger spatial orienting to socially relevant information rather than arrow cues, as reflected in both early (N1) and late (N2 and P3b) stages.

## Figures and Tables

**Table 1:** Mean RTs and ACC in Experiment 1.

Cue Type	Cue Validity	Target Type	RT (ms)		ACC (%)	
			*M*	*SE*	*M*	*SE*
Arrow cue	valid	threatening	616	11.94	91.4	1.14
		neutral	592	12.54	96.6	0.62
	invalid	threatening	636	13.43	91.8	0.82
		neutral	601	12.01	96.5	0.64
Gaze cue	valid	threatening	618	12.96	92.0	1.15
		neutral	604	12.51	96.6	0.86
	invalid	threatening	624	12.27	92.7	0.98
		neutral	603	11.89	96.9	0.51

## Data Availability

All data and materials have been made publicly available via the Open Science Framework and can be accessed at https://osf.io/dhb74/.

## Appendix A

**Table A1:** List of target stimuli.

Neutral Targets		Threatening Targets	
Small	Big	Small	Big
Book	Refrigerator	Poisoned bottle	Tank
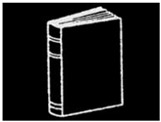
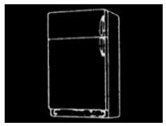
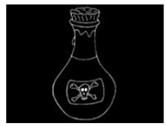
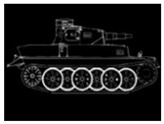
Brush	Chair	Broken beer bottle	Saw
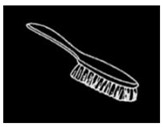
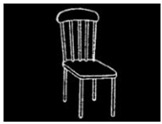
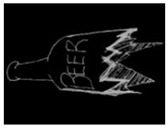
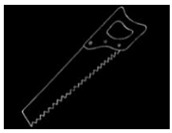
Envelope	Church	Shuriken	Sabre
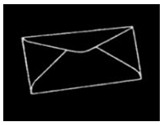
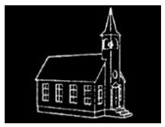
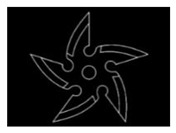
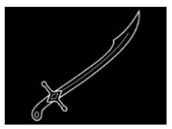
Shoe	Rocking chair	Boomerang	Sword
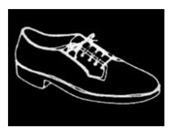
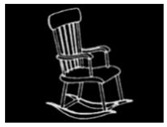
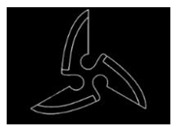
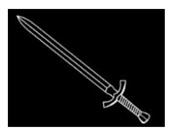
Key	Table	Pistol	Assault rifle
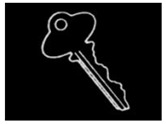
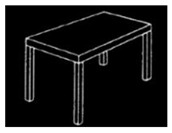
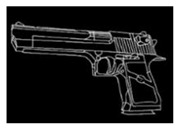
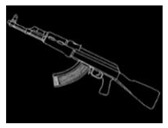
Lock	Television	Revolver	Sniper rifle
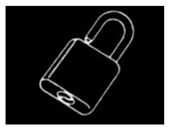
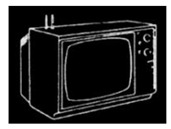
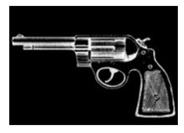
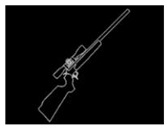
Whistle	Dresser	Dagger	Assault rifle
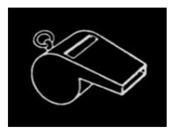
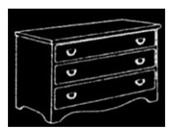
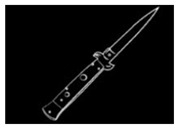
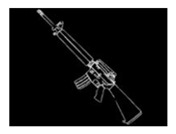
Watch	Ironing board	Knife	Scimitar

## References

[1] Huang C.-M., Andrist S., Sauppé A., Mutlu B. (2015). Using gaze patterns to predict task intent in collaboration. Front. Psychol..

[2] Morgan E.J., Freeth M., Smith D. (2018). Mental State Attributions Mediate the Gaze Cueing Effect. Vision.

[3] Emery N.J. (2000). The eyes have it: The neuroethology, function and evolution of social gaze. Neurosci. Biobehav. Rev..

[4] Siposova B., Carpenter M. (2019). A new look at joint attention and common knowledge. Cognition.

[5] McKay K.T., Grainger S.A., Coundouris S.P., Skorich D.P., Phillips L.H., Henry J.D. (2021). Visual attentional orienting by eye gaze: A meta-analytic review of the gaze-cueing effect. Psychol. Bull..

[6] Brooks R., Meltzoff A.N. (2005). The development of gaze following and its relation to language. Dev. Sci..

[7] Charman T., Baroncohen S., Swettenham J., Baird G., Cox A., Drew A. (2000). Testing joint attention, imitation, and play as infancy precursors to language and theory of mind. Cogn. Dev..

[8] Rayson H., Bonaiuto J.J., Ferrari P.F., Chakrabarti B., Murray L. (2019). Building blocks of joint attention: Early sensitivity to having one’s own gaze followed. Dev. Cogn. Neurosci..

[9] Posner M.I. (1980). Orienting of attention. Q. J. Exp. Psychol..

[10] Driver J., Davis G., Ricciardelli P., Kidd P., Maxwell E., Baron-Cohen S. (1999). Gaze Perception Triggers Reflexive Visuospatial Orienting. Vis. Cogn..

[11] Friesen C.K., Kingstone A. (1998). The eyes have it! Reflexive orienting is triggered by nonpredictive gaze. Psychon. Bull. Rev..

[12] Tipples J. (2002). Eye gaze is not unique: Automatic orienting in response to uninformative arrows. Psychon. Bull. Rev..

[13] Besner D., McLean D., Young T. (2021). Do eyes and arrows elicit automatic orienting? Three mutually exclusive hypotheses and a test. Q. J. Exp. Psychol..

[14] Breil C., Huestegge L., Böckler A. (2022). From eye to arrow: Attention capture by direct gaze requires more than just the eyes. Atten. Percept. Psychophys..

[15] Friesen C.K., Ristic J., Kingstone A. (2004). Attentional effects of counterpredictive gaze and arrow cues. J. Exp. Psychol. Hum. Percept. Perform..

[16] Hietanen J.K., Leppänen J.M., Nummenmaa L., Astikainen P. (2008). Visuospatial attention shifts by gaze and arrow cues: An ERP study. Brain Res..

[17] Langdon R., Smith P. (2005). Spatial cueing by social versus nonsocial directional signals. Vis. Cogn..

[18] Ristic J., Wright A., Kingstone A. (2007). Attentional Control and Reflexive Orienting to Gaze and Arrow Cues. Psychon. Bull. Rev..

[19] Slessor G., Finnerty A., Papp J., Smith D., Martin D. (2019). Gaze-cueing and endogenous attention operate in parallel. Acta Psychol..

[20] Galfano G., Dalmaso M., Marzoli D., Pavan G., Coricelli C., Castelli L. (2012). Eye gaze cannot be ignored (but neither can arrows). Q. J. Exp. Psychol..

[21] Guzzon D., Brignani D., Miniussi C., Marzi C.A. (2010). Orienting of attention with eye and arrow cues and the effect of overtraining. Acta Psychol..

[22] Marotta A., Casagrande M., Lupiáñez J. (2013). Object-based attentional effects in response to eye-gaze and arrow cues. Acta Psychol..

[23] Brignani D., Guzzon D., Marzi C.A., Miniussi C. (2009). Attentional orienting induced by arrows and eye-gaze compared with an endogenous cue. Neuropsychologia.

[24] Tipper C., Handy T.C., Giesbrecht B., Kingstone A. (2008). Brain responses to biological relevance. J. Cogn. Neurosci..

[25] Aranda-Martín B., Ballesteros-Duperón M.Á., Lupiáñez J. (2022). What gaze adds to arrows: Changes in attentional response to gaze versus arrows in childhood and adolescence. Br. J. Psychol..

[26] Marotta A., Lupiáñez J., Martella D., Casagrande M. (2012). Eye gaze versus arrows as spatial cues: Two qualitatively different modes of attentional selection. J. Exp. Psychol. Hum. Percept. Perform..

[27] Lassalle A., Itier R.J. (2015). Emotional modulation of attention orienting by gaze varies with dynamic cue sequence. Vis. Cogn..

[28] McCrackin S.D., Itier R.J. (2019). Individual differences in the emotional modulation of gaze-cuing. Cogn. Emot..

[29] Zhang J., He X., Sommer W., Yue Z. (2021). Does gaze direction of fearful faces facilitate the processing of threat? An ERP study of spatial precuing effects. Cogn. Affect. Behav. Neurosci..

[30] Dalmaso M., Castelli L., Galfano G. (2020). Social modulators of gaze-mediated orienting of attention: A review. Psychon. Bull. Rev..

[31] Marotta A., Román-Caballero R., Lupiáñez J. (2018). Arrows don’t look at you: Qualitatively different attentional mechanisms triggered by gaze and arrows. Psychon. Bull. Rev..

[32] Marotta A., Lupiáñez J., Román-Caballero R., Narganes-Pineda C., Martín-Arévalo E. (2019). Are eyes special? Electrophysiological and behavioural evidence for a dissociation between eye-gaze and arrows attentional mechanisms. Neuropsychologia.

[33] Yan T., Zhao S., Uono S., Bi X., Tian A., Yoshimura S., Toichi M. (2016). Target object moderation of attentional orienting by gazes or arrows. Atten. Percept. Psychophys..

[34] Zhao S., Uono S., Yoshimura S., Toichi M. (2014). Attention orienting by eye gaze and arrows reveals flexibility to environmental changes. Acta Psychol..

[35] Fox E., Griggs L., Mouchlianitis E. (2007). The detection of fear-relevant stimuli: Are guns noticed as quickly as snakes?. Emotion.

[36] Ohman A., Flykt A., Esteves F. (2001). Emotion drives attention: Detecting the snake in the grass. J. Exp. Psychol. Gen..

[37] Ishikawa M., Haensel J.X., Smith T.J., Senju A., Itakura S. (2021). Affective priming enhances gaze cueing effect. J. Exp. Psychol. Hum. Percept. Perform..

[38] Jescheniak J.D., Schriefers H., Garrett M.F., Friederici A.D. (2002). Exploring the activation of semantic and phonological codes during speech planning with event-related brain potentials. J. Cogn. Neurosci..

[39] Panichello M.F., Kveraga K., Chaumon M., Bar M., Barrett L.F. (2017). Internal valence modulates the speed of object recognition. Sci. Rep..

[40] Schneider T.R., Engel A.K., Debener S. (2008). Multisensory identification of natural objects in a two-way crossmodal priming paradigm. Exp. Psychol..

[41] Lassalle A., Itier R.J. (2015). Autistic traits influence gaze-oriented attention to happy but not fearful faces. Soc. Neurosci..

[42] Kenemans J.L., Kok A., Smulders F.T. (1993). Event-related potentials to conjunctions of spatial frequency and orientation as a function of stimulus parameters and response requirements. Electroencephalogr. Clin. Neurophysiol..

[43] Potts G.F., Patel S.H., Azzam P.N. (2004). Impact of instructed relevance on the visual ERP. Int. J. Psychophysiol..

[44] Luck S.J., Woodman G.F., Vogel E.K. (2000). Event-related potential studies of attention. Trends Cogn. Sci..

[45] Breton F., Ritter W., Simson R., Vaughan H.G. (1988). The N2 component elicited by stimulus matches and multiple targets. Biol. Psychol..

[46] Folstein J.R., Van Petten C. (2008). Influence of cognitive control and mismatch on the N2 component of the ERP: A review. Psychophysiology.

[47] Miller R.K., Martin F.H. (2020). Deconstructing threat: Rethinking the interplay between biological and social relevance in the emotional salience of unpleasant images. Biol. Psychol..

[48] Willis M.L., Palermo R., Burke D., Atkinson C.M., McArthur G. (2010). Switching associations between facial identity and emotional expression: A behavioural and ERP study. NeuroImage.

[49] March D.S., Gaertner L., Olson M.A. (2017). In Harm’s Way: On Preferential Response to Threatening Stimuli. Personal. Soc. Psychol. Bull..

[50] Faul F., Erdfelder E., Buchner A., Lang A.G. (2009). Statistical power analyses using G*Power 3.1: Tests for correlation and regression analyses. Behav. Res. Methods.

[51] Langner O., Dotsch R., Bijlstra G., Wigboldus D.H.J., Hawk S.T., van Knippenberg A. (2010). Presentation and validation of the Radboud Faces Database. Cogn. Emot..

[52] Weldon M.S., Roediger H.L. (1987). Altering retrieval demands reverses the picture superiority effect. Mem. Cogn..

[53] Bell A.J., Sejnowski T.J. (1995). An information-maximization approach to blind separation and blind deconvolution. Neural Comput..

[54] Hong X., Sun J., Wang J., Li C., Tong S. (2020). Attention-related modulation of frontal midline theta oscillations in cingulate cortex during a spatial cueing Go/NoGo task. Int. J. Psychophysiol..

[55] Wascher E., Hoffmann S., Sänger J., Grosjean M. (2009). Visuo-spatial processing and the N1 component of the ERP. Psychophysiology.

[56] Ma J., Liu C., Chen X. (2016). Emotional Modulation of Conflict Processing in the Affective Domain: Evidence from Event-Related Potentials and Event-Related Spectral Perturbation Analysis. Sci. Rep..

[57] Pan D.N., Wang Y., Lei Z., Wang Y., Li X. (2019). The altered early components and the decisive later process underlying attention bias modification in social anxiety: Evidence from event-related potentials. Soc. Cogn. Affect. Neurosci..

[58] Engell A.D., Nummenmaa L., Oosterhof N.N., Henson R.N., Haxby J.V., Calder A.J. (2010). Differential activation of frontoparietal attention networks by social and symbolic spatial cues. Soc. Cogn. Affect. Neurosci..

[59] Hietanen J.K., Nummenmaa L., Nyman M.J., Parkkola R., Hämäläinen H. (2006). Automatic attention orienting by social and symbolic cues activates different neural networks: An fMRI study. NeuroImage.

[60] Azizian A., Freitas A.L., Watson T.D., Squires N.K. (2006). Electrophysiological correlates of categorization: P300 amplitude as index of target similarity. Biol. Psychol..

[61] Johnson R., Donchin E. (1980). P300 and stimulus categorization: Two plus one is not so different from one plus one. Psychophysiology.

[62] Kutas M., McCarthy G., Donchin E. (1977). Augmenting mental chronometry: The P300 as a measure of stimulus evaluation time. Science.

[63] Tipples J. (2006). Fear and fearfulness potentiate automatic orienting to eye gaze. Cogn. Emot..

[64] Baskin-Sommers A.R., Curtin J.J., Newman J.P. (2013). Emotion-modulated startle in psychopathy: Clarifying familiar effects. J. Abnorm. Psychol..

[65] Zsido A.N., Deak A., Bernath L. (2019). Is a snake scarier than a gun? The ontogenetic-phylogenetic dispute from a new perspective: The role of arousal. Emotion.

[66] Bayliss A.P., Frischen A., Fenske M.J., Tipper S.P. (2007). Affective evaluations of objects are influenced by observed gaze direction and emotional expression. Cognition.

[67] Bayliss A.P., Schuch S., Tipper S.P. (2010). Gaze cueing elicited by emotional faces is influenced by affective context. Vis. Cogn..

[68] Chen Z., McCrackin S.D., Morgan A., Itier R.J. (2021). The gaze cueing effect and its enhancement by facial expressions are impacted by task demands: Direct comparison of target localization and discrimination tasks. Front. Psychol..

[69] Jaswal S., Logie R.H. (2013). The contextual interference effect in visual feature binding: What does it say about the role of attention in binding?. Q. J. Exp. Psychol..

